# Ancillary human health benefits of improved air quality resulting from climate change mitigation

**DOI:** 10.1186/1476-069X-7-41

**Published:** 2008-07-31

**Authors:** Michelle L Bell, Devra L Davis, Luis A Cifuentes, Alan J Krupnick, Richard D Morgenstern, George D Thurston

**Affiliations:** 1 School of Forestry and Environmental Studies, Yale University, New Haven, CT 06511, USA; 2 Graduate School of Public Health, University of Pittsburgh, CNPAV 435, Pittsburgh, PA 15260, USA; 3 Industrial and Systems Engineering Department, P. Catholic University of Chile, Engineering School, Santiago, Chile; 4 Resources for the Future, Washington, DC 20036, USA; 5 School of Medicine, New York University, Tuxedo, NY 10987, USA

## Abstract

**Background:**

Greenhouse gas (GHG) mitigation policies can provide ancillary benefits in terms of short-term improvements in air quality and associated health benefits. Several studies have analyzed the ancillary impacts of GHG policies for a variety of locations, pollutants, and policies. In this paper we review the existing evidence on ancillary health benefits relating to air pollution from various GHG strategies and provide a framework for such analysis.

**Methods:**

We evaluate techniques used in different stages of such research for estimation of: (1) changes in air pollutant concentrations; (2) avoided adverse health endpoints; and (3) economic valuation of health consequences. The limitations and merits of various methods are examined. Finally, we conclude with recommendations for ancillary benefits analysis and related research gaps in the relevant disciplines.

**Results:**

We found that to date most assessments have focused their analysis more heavily on one aspect of the framework (e.g., economic analysis). While a wide range of methods was applied to various policies and regions, results from multiple studies provide strong evidence that the short-term public health and economic benefits of ancillary benefits related to GHG mitigation strategies are substantial. Further, results of these analyses are likely to be underestimates because there are a number of important unquantified health and economic endpoints.

**Conclusion:**

Remaining challenges include integrating the understanding of the relative toxicity of particulate matter by components or sources, developing better estimates of public health and environmental impacts on selected sub-populations, and devising new methods for evaluating heretofore unquantified and non-monetized benefits.

## Background

Averting the course of climate change would result in human health benefits directly associated with lessened global temperature changes and associated impacts, but would also bring ancillary health benefits from reduced ground-level air pollution in the short-term [1-5]. Many fossil-fuel combustion processes that generate greenhouse gases (GHG) also emit other harmful air pollutants. Several measures aimed at reducing GHG emissions can also improve local air quality, most commonly particulate matter (PM) and ozone (O_3_) precursors. Further, whereas the benefits from climate change mitigation would materialize far in the future, co-benefits, or ancillary benefits, would occur in the short-term.

**Figure 1:**
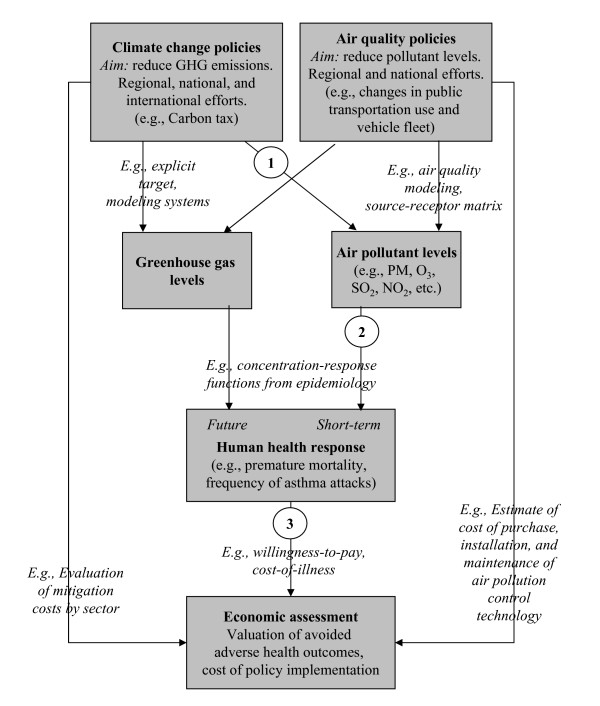
Relationship between climate change and air quality policies.

Figure 1 describes the relationships among the health consequences of climate change and air quality policies and the general framework of how these responses can be assessed. Air quality policies are routinely evaluated in terms of the estimated health outcomes avoided and their economic impact [6,7]. However, assessment of the health impacts of GHG strategies often considers only consequences in the far future (i.e., left side of Figure 1), without integration of the short-term benefits of related policies [8]. Well-informed public health and environmental strategies require full consideration of consequences, including co-benefits and potential ancillary harms.

A broad array of tools to evaluate the health-related ancillary costs and benefits of climate change is currently available, and some examples are provided in italics in Figure 1. The general structure for most assessments involves three key steps: (1) estimating changes in air pollutant concentrations, comparing levels in response to GHG mitigation to concentrations under a baseline "business-as-usual" scenario; (2) estimating the adverse health impacts avoided from reduced air pollution; and (3) for some studies, estimating the monetary benefit from these averted health consequences, often with comparison to the cost of the climate change mitigation measure. The first step is sometimes accomplished through emissions scenarios and information regarding how emissions translate into pollutant concentrations, such as with air quality modeling systems. The second step usually relies on concentration-response functions from existing epidemiological studies on ambient air pollution and health. The third stage utilizes a variety of techniques to translate health benefits into monetary terms, such as contingent valuation (CV). Additional steps include sensitivity analysis, such as applying multiple climate change scenarios or concentration-response functions for health effects.

This paper aims to illuminate the weight of evidence on the ancillary health benefits of GHG policies, provide a framework for such analysis, and critique relevant methods. We focus on the effects of air quality; however a full assessment of the complete ancillary consequences would consider other factors such as the cost of mitigation measures and ecological impacts. We close with recommendations on the appropriate role of ancillary health benefits and costs in the climate change mitigation debate. As part of these recommendations, we identify a number of public health and economic related research topics that require clarification in order to promote more effective ancillary benefits assessments with respect to GHG mitigation policies.

### Studies of ancillary benefits

A variety of studies have been conducted to estimate the health and air pollution ancillary benefits from GHG reduction, with a wide range of methods and study areas. Energy scenarios, emission inventories, and global change and regional air quality modeling systems have been linked to estimate the short-term incremental changes in public health and the environment that could result from various GHG mitigation policies [9,10].

Recently, the Stern Review [11] addressed a wide range of global benefits and costs associated with climate change, including air pollution co-benefits. Citing a study by the European Environmental Agency, the Review notes that limiting global mean temperature increase to 2°C would lead to annual savings in the implementation of existing European air pollution control measures of €10 billion and additional avoided annual health costs of €16–46 billion. Even larger co-benefits are estimated in developing countries, including via the substitution of modern fuels for biomass. The Stern Review also recognizes some of the trade-offs between climate change objectives and local air quality gains. For instance, switching from petrol to diesel reduces carbon dioxide (CO_2_) emissions but increases particles with aerodynamic diameter ≤ 10 μm (PM_10_) and nitrogen oxides (NO_x_) emissions. Increasing combustion temperatures of aircraft engines reduces CO_2 _while increasing NO_x_, as well as water vapor, which can intensify warming effects. Other GHG mitigating actions present fewer environmental trade-offs (e.g., reductions in aircraft weight can decrease CO_2 _emissions and simultaneously improve local air quality).

A study of three Latin American cities identified significant health benefits from reducing GHG, including about 64,000 cases of avoided premature mortality over a 20-year period [12]. Reducing methane concentrations by 20% starting in 2010 was estimated to lower tropospheric O_3 _levels, averting over 30,000 deaths worldwide in 2030 alone [13]. Country-wide assessments of GHG mitigation policies on public health have been produced for Canada [14] and selected energy sectors in China [15,16], under differing baseline assumptions. A synthesis of research on co-benefits and climate change policies in China concluded that China's Clean Development Mechanism potentially could save 3,000–40,000 lives annually through co-benefits of improved air pollution [17]. Several studies investigated the links between regional air pollution and climate policy in Europe [18-20]. The table in additional file 1 summarizes key examples of co-benefits studies and briefly describes the methods used for each step of analysis.

Results from co-benefits studies are typically difficult to compare, even if study area and target year are identical, due to variations in study design. Major differences exist in the methodology used to estimate benefits, as demonstrated in the table in additional file 1. Whereas some studies implement sophisticated modeling systems to estimate altered air quality, capturing regional differences in pollutant levels [13], others use simple target values with uniform pollution reductions across all spatial areas [12]. Likewise, some studies estimate changes in health impacts based on a single or small number of concentration-response functions, capturing only a portion of the health impacts and at times assuming that concentration-response functions derived from one area are applicable in others [21], while other analyses select locally developed concentration-response functions where available and consider a wide range of health impacts [12]. Each approach depends on different underlying conjectures. Even with the widely varying methods, results consistently indicate significant ancillary health benefits from GHG policies. Similarly, estimates of the social cost of air pollution policies were found to be quite insensitive to choices in the uncertainties of costs and benefits [22].

### Estimation of changes in air pollutant concentrations

Reductions in local air pollutants resulting from GHG policies (step 1 in Figure 1) can be calculated based on the resulting pollutant levels under a baseline and climate mitigation scenarios. Research designs differ not only by the policy studied but the choice of a baseline "business-as-usual" scenario. Options range from assumptions that emissions or pollutant concentrations remain at current levels, perhaps adjusted for population growth, to aggressive air pollution control policies regardless of actions taken to affect GHGs. A review of studies of ancillary benefits concentrating on the energy sector found that choice of baseline scenario greatly impacted results, especially for studies assuming lower pollution levels as directed by the 1990 Clean Air Act Amendments (CAAA) in comparison to those omitting the CAAA [23].

Uncertainties in climate change predictions and estimation of regional parameters can be considerable, especially for highly disaggregated assessments with long-term projections [24-26]. However, assessment of ancillary benefits requires estimates of pollution levels a few years into the future, not several decades, and thus is not marred by uncertainties that plague many other forms of climate-related research. The longest projections for studies in Additional file 1 are about 20 years.

Approaches to estimate changes in air pollution range from complex modeling systems to a simple pollution target, assuming a pollutant's levels will be at a specified concentration or meet a certain absolute or relative reduction by a given date. Existing emissions inventories and source-receptor matrices can be used to connect changes in emissions to changes in specific pollutants [27-31]. Backwards trajectory modeling has been used to determine pollutant sources and locations [32-36], and this information can then be used to estimate how changes in pollutant emissions will affect concentrations at various locales. Regional air quality modeling systems, such as the USEPA's Models-3/Community Multi-Scale Air Quality (CMAQ) model in conjunction with meteorological models, link data on meteorology, emissions, and land-use to generate gridded estimates of pollutants, including O_3 _and PM at various size fractions [37]. Such modeling has been used to estimate how changes in emissions scenarios affect ambient concentrations [38,39] and similarly can be applied to estimate future changes in ambient pollutants from climate change measures [40] and future impacts on human health [41].

The choice of method to ascertain future pollutant levels depends on what pollutants and regions are considered and the spatial and temporal resolution desired. For example, a recent study [13] applied a sophisticated air quality modeling system to estimate O_3 _levels across different regions of the world, whereas other approaches [12,42] applied uniform reductions assuming equal percent reductions in pollutants across all areas. The more advanced approach using modeling systems is better equipped to capture spatial variability and transport of pollution and precursors; however some pollutants are more easily modeled than others.

Uncertainties in the translation of a given climate policy to changes in pollutant concentrations vary by the method used, but include: (1) the choice of "baseline" scenario; (2) translation of a policy into emissions changes in various sectors; (3) physical transformation of the pollutant (e.g., agglomeration of particles to a larger size); (4) chemical transformation of pollutants (e.g., non-linear transformation of O_3 _precursors, conversion of gaseous pollutants such as NO_x _to particles); and (5) spatial and temporal distribution of impacts, as a function of the preceding factors. Both the baseline scenario and climate change mitigation policies are assumed to have uniform or otherwise known spatial and temporal distribution in pollution levels. This can be particularly important if emissions trading is included, such as SO_2 _cap and trade programs, which set a maximum value for emissions but allow large heterogeneity in emissions that can change with time. The level of uncertainty may differ by pollutant depending on their spatial heterogeneity. For example, within-city gradients have been observed for PM_2.5 _[43]. While most ancillary studies to date have examined policies at the federal level, in theory analysis could examine the impacts of other mitigation actions such as those conducted at the local level [44,45] or even personal choice and household level actions [46,47] that aggregated lead to lower GHG emissions.

### Estimation of human health impacts

Studies of the health effects potentially avoidable by climate change mitigation strategies have been based almost exclusively on concentration-response functions derived from published epidemiological studies (step 2 of Figure 1). Common urban air pollutants likely to be impacted by GHG policy (e.g., PM) have been associated with a wide range of harmful health impacts including increased frequency of hospital admissions and increased risk of mortality [48]. Table 1 provides the health outcomes and sources of concentration-response coefficients employed for the subset of studies in additional file 1 that estimated health impacts. Because mortality dominates benefits analyses, additional detail is given on the pollutants and timeframe of exposure (i.e., acute or chronic) for mortality.

**Table 1:** Concentration-response functions used in the assessments listed in Additional file 1

**Mortality**	**Morbidity**
**Aaheim et al. 1999 **[153]	
PM: Adult and infant [160]	Lung-cancer, acute and chronic respiratory symptoms, pseudo-croup, asthma [160]

**Aunan et al. 2004 **[75]	
PM_10 _(chronic): modified version of Pope et al. 1995 [160]	Outpatient visits, emergency room visits, hospital admissions, work loss days, acute respiratory symptoms in children and adults, chronic respiratory symptoms in children and adults, asthma attacks [161]

**Burtraw et al. 2003 **[23]	
PM_10 _and nitrates (acute) [162]	NO_x_: respiratory symptoms, eye irritation days, phlegm days [163]

**Cifuentes et al. 2001 **[12,42]	
PM_10 _(acute and chronic):	Respiratory hospital admissions [171,172], emergency department visits [173], chronic adult bronchitis [174], acute bronchitis in children [56], asthma attacks [175], work loss days [176], restricted activity days (RAD) [177-179], respiratory symptom days [180]
Adults [164-169]	
Infants [84,170]	
O_3 _(acute) [50]	

**Dessus and O'Connor 2003 **[155]	
PM_10 _(acute): Based on previously conducted literature reviews [181]	Respiratory hospital admissions, emergency room visits, RAD, MRAD, clinic visits for bronchitis for children <15 years, respiratory symptoms for adults and children, chronic bronchitis, chest discomfort, eye irritation, headaches. Based on previously conducted literature reviews [181,182]

**Dudek et al. 2003 **[156]	
Did not apply concentration-response functions. Estimated changes in mortality based on baseline burden.	Respiratory disease and neoplasm. Did not apply concentration-response functions. Estimated changes in morbidity based on baseline burden.

**Mazzi and Dowlatabadi 2007 **[157]	
PM_2.5 _(chronic) [167,183,184]	Respiratory and cardiovascular (CVD) hospitalizations [185]

**McKinley et al. 2005 **[49]	
PM_10 _(acute and chronic) [167,183,186] O_3 _(acute) [187]	Chronic bronchitis [188], MRAD [176,178], emergency room visits and hospital admissions: previously conducted review [189]

**Wang and Smith 1999 **[15]	
PM (acute and chronic) [190-192]	Respiratory hospital admissions [193], emergency room visits [194], RAD >16 years [177], acute bronchitis <16 years [195], asthma attacks per asthmatic [175,196], respiratory symptoms [180], chronic bronchitis >16 years [174]

**West et al. 2006 **[13]	
O_3 _(acute) [51]	

*Note: *References for health endpoints refer to the concentration-response function applied.

In this context, the method involves applying a mathematical relationship between pollution levels associated with various types of health endpoints, with an understanding of the relationships between the health effect and individual (or social) preferences for reducing the risk or incidence of this effect. The use of a concentration-response function without adjustment assumes that the underlying relationship between air pollution and health when and where the function was derived will hold in the future, perhaps in a different location. This integration involves matching as closely as possible the starting point of the valuation analysis to the endpoint provided by health science, that is a measure of pollution (e.g., ambient levels as a surrogate for exposure) to a health response (e.g., increased risk in hospitalization). In addition, the approach requires knowledge of the population by cohorts that map to the health endpoints (e.g., asthmatics or those >65 years) and assumptions regarding baseline health responses.

Critical differences in this stage of analysis are choice of pollutants, health effects, time scale (e.g., acute versus chronic), epidemiological studies, and assumptions (e.g., baseline mortality rate). Almost all studies in Table 1 estimated averted mortality for PM, however a variety of exposure-response coefficients were used, and several studies made assumptions regarding conversion of one pollution form to another (e.g., equal toxicity for nitrates and PM_10 _[23], PM_2.5_/PM_10 _= 0.6 [49], PM_10_/TSP = 0.5 [15]).

Criteria for selection of health endpoints and epidemiological studies were not consistent across the studies, however common themes were: (1) use of locally conducted studies where possible; (2) health endpoints with a consistent literature demonstrating a relationship with air pollution; and (3) emphasis on peer-reviewed research, although some studies applied non-peer-reviewed work. As the epidemiological literature grows, integrated assessments that incorporate these findings also evolve. For example, earlier studies estimating averted mortality from lowered O_3 _levels were based on epidemiological research of a single city (e.g., a Los Angeles study [50] applied to Latin America [12,42]), whereas more recent work uses multi-city epidemiological studies to generate global estimates (e.g., a 95-city study [51] applied worldwide [13]). Concentration-response functions derived from numerous cities have advantages over single-city studies as they are less subject to sample size concerns and city-specific results can be combined to generate an overall estimate accounting for within-city and between-city statistical uncertainty [52]. The choice of location of the epidemiological studies used may be based on selecting a city or region matching or similar to that of the ancillary benefits assessment. If only non-local single-city studies are available, options are to perform a meta-analysis to generate an average that accounts for the uncertainty of each city-specific relative rate or to select an existing multi-city study.

An alternative to identifying epidemiological studies through literature review is to apply an existing database or model of concentration-response functions, many of which also include economic valuation tools. The Fast Environmental Regulatory Evaluation Tool (FERET) is a cost-benefit template developed by Carnegie Mellon University and the University of Washington to evaluate how policy changes affect air-related health outcomes and their associated economic impacts [53]. The Benefits Mapping and Analysis Program (BenMAP), developed by USEPA, estimates population-level exposures, changes in health endpoints, and economic values [54]. The Ozone Risk Assessment Model (ORAM) uses air quality modeling to predict changes in O_3 _levels and associated health response [55]. These systems can be used to estimate changes in health and their monetary value, or as a source of concentration-response and economic value functions [55-57].

A number of key uncertainties characterizes the use of population-based research on air pollution and health for ancillary benefits studies. These include [58,59]:

#### Causality

The precise physiological mechanism(s) by which air pollution could cause the health effects indicated in epidemiologic studies is not always fully understood. As a result causal inferences are generally developed based on consistent evidence across multiple epidemiological studies including different areas and study designs, and results from toxicological and human exposure studies in conjunction with the criteria of biological plausibility.

#### Other pollutants and pollutant mixtures

Often co-pollutants are included in integrated assessments separately and their health or economic consequences summed. This may underestimate or overestimate actual damages. The true harmful agent may not be the pollutant under study but a related pollutant or group of pollutants with similar sources and/or formation pathways. For example, O_3 _can be considered a marker for an array of photochemical pollutants. Nitrates and sulfates are related to PM as they contribute to secondary particles. Interaction between multiple pollutants is not well understood, and most results are presented for an individual pollutant, although air pollution is experienced as a mixture.

#### Toxicity relating to PM chemical composition

While a substantial literature provides consistent evidence that particles are detrimental to health and a limited number of population-based studies have examined PM effects by chemical composition [60], the differential toxicity of various forms of the PM mixture is unidentified. Differential effects have been demonstrated based on particle size, however chemical composition also appears to play a role as the same size distribution provides different effect estimates based on region [61,62]. In current analysis of ancillary benefits, all particles of a given size (e.g., PM_2.5_) may be treated with equivalent toxicity, however if for example sulfates are more harmful than other particles, technologies that reduce emissions of particles from coal combustion may result in greater health benefits than other technologies. If, for example, elemental carbon is identified to be more detrimental to health, transportation technologies may be more effective.

#### Use of ambient monitors

The vast majority of epidemiological studies applied in ancillary benefits studies use ambient monitoring data as a surrogate for individual or community-level exposure. The relationship between personal exposure and ambient monitoring data varies by pollutant, typically with better correlation for particles than for O_3 _[63,64]. Use of ambient monitors increases the possibility of exposure misclassification, which if non-differential would generally drive effect estimates towards the null, resulting in underestimates. This issue has particular importance for the extrapolation of concentration-response functions from one area to another, as the relationship between ambient monitors and exposure, and thereby health, is a function of indoor pollution and indoor/outdoor activity patterns, which may vary widely across populations.

#### Shape of concentration-response functions

Many concentration-response functions applied in ancillary benefits studies assume a log-linear relationship between exposure and risk. If the true shape differs, incorrect estimates could be obtained. If the assessment includes pollutant levels above those used to generate the concentration-response function, results will be distorted if the log-linear or otherwise assumed function does not hold. If there exists a safe level below which pollution does not adversely impact health, calculations based on functions assuming no threshold would be incorrect for pollutant levels below the threshold value. Some studies have examined the shape of the concentration-response curve, however such analysis does not exist for all pollutants and health outcomes. Several recent US-based studies found no evidence of a threshold at typical concentrations for the relationship between mortality and O_3 _[65] or PM [66].

#### Temporal or spatial extrapolation

Pollution and health relationships developed in one area may not be applicable in another location due to differences in the underlying population and pollutant characteristics [67]. Efforts are often made to apply locally-derived studies [49], however concentration-response functions do not exist for many outcomes and pollutants for much of the world. Therefore US and European studies are generally employed, although a growing number of epidemiological studies are underway in Asia and Latin America [68-71]. Uncertainties introduced by such extrapolation include differences in indoor/outdoor activity patterns, population characteristics, household characteristics that relate to exposure, and the pollution mixture. Likewise, the study of ancillary benefits involves future societies that may have dissimilar housing, populations, health care systems, and pollutant mixtures compared to the present day or the timeframe of the epidemiological research.

#### Chronic and acute effects and exposures

Air pollution exposure can be categorized as short-term (i.e., a few days) or longer term (i.e., a few months or years). Health impacts can be classified as those that take place immediately or a short time after exposure, or those that have a gradual or much-delayed response, such as cancer and neurological disease. Cohort studies of PM, which evaluate long-term exposure, generally provide higher estimates for mortality than do time-series studies, which evaluate short-term exposure [72,73]. Often more information is available regarding health impacts of short-term exposure because such exposure estimates are more readily available. However, the use of only acute-exposure impacts may underestimate the total mortality burden from air pollution [74]. Co-benefits studies have used different approaches to address chronic and acute health impacts. Whereas one study [75] included estimates of chronic mortality, excluding acute mortality effects, another [23] incorporated acute mortality only.

#### Unknown health endpoints

While air pollution has been quantitatively linked to many health consequences, there are other health events, including several pediatric and neurological endpoints, for which concentration-response functions have not yet been developed. Some of these health responses are less severe than the more commonly studied effects. However as a counter example, recent studies elucidated the link between O_3 _and mortality [51,76-78]. Although less severe health endpoints have lower monetary valuations than more severe impacts, they often occur in larger numbers. Thus, the more grave outcomes such as death and hospital admissions are best viewed as indicators of the much broader spectrum of adverse health effects resulting from air pollution.

#### Degree of mortality displacement

The public health burden of mortality associated with air pollution depends not only on the increased risk of death, but also on the length of life shortening. Several recent studies provide evidence that short-term mortality displacement of a few days or less does not account for the observed PM mortality effect estimates [79-83]. Past evaluations of air pollution's effect on life expectancy generally considered only deaths among adults above 30 years of age, but some studies [84-86] suggest that infants may be among the sub-populations particularly affected by long-term PM exposure, which would indicate a much larger reduction in life expectancy. Currently, considerable uncertainty remains as to the amount of life-shortening associated with air pollution.

### Economic valuation of avoided adverse health outcomes

To help decision-makers assess policies with a wide array of consequences, outcomes are often converted into comparable formats. Several multi-criteria decision-making techniques have been applied in the context of climate change policy [87-90]. Another widely used approach is to convert health outcomes into economic terms to allow direct comparison of costs and benefits. Underlying economic valuation of health is the concept that individuals have preferences that extend over environmental quality, market goods, and other non-market goods. If this assumption is accepted, in principle it is possible to deduce how individuals tradeoff health by measuring how much in the way of other services individuals are willing to forego to enjoy health benefits. Expression of these values in monetary terms is used as a surrogate for what people are willing to give up in alternative real consumption opportunities. The notion that such individual tradeoffs well describe society's interest in environmental quality is by no means universally accepted, and controversy surrounds economic valuation and benefit-cost analysis in particular [91]. For a summary of the economic argument see [92].

#### Approaches for economic valuation of health

We identified several approaches for economic valuation of averted health consequences (step 3 of Figure 1): COI; human capital; a variety of WTP methods; and quality-adjusted life year (QALY) approaches.

#### Cost of illness

The COI method totals medical and other out-of-pocket expenditures and has been used for acute and chronic health endpoints. For instance, separate models of cancer progression and respiratory disease were used to estimate medical costs from these diseases over one's lifetime [93]. COI incorporates direct medical costs, such as for physicians' visits and medications, and indirect costs, including lost income from work loss days. However the approach does not capture other consequences of illness such as psychological suffering, physical pain, transportation to medical appointments, dietary restrictions, and expenditures for friends or family acting as caretakers. The approach can have a welfare theoretic basis, but does not reflect the full damage of illness, hence results usually underestimate costs and should be considered a lower bound. Some COI studies assign a medical expenditure based on primary diagnosis [94].

#### Human capital approach

Early attempts to value mortality risk reductions applied the human capital approach, which estimates the "value of life" as lost productivity. This method is generally recognized as problematic and not based on modern welfare economics, where preferences for reducing death risks are not captured. Another limitation is incorporation of racial- or gender-based discrimination in wages. This method assigns value based solely on income, without regard to social value, so unpaid positions such as homemaker and lower paid positions such as social worker receive lower values. Because data are often available for superior alternatives, this approach is rarely used in health benefit studies.

#### Willingness to pay

WTP generates estimates of preferences for improved health that meet the theoretical requirements of neoclassical welfare economics, by aiming to measure the monetary amount persons would willingly sacrifice to avoid negative health outcomes. Complications arise in analysis and interpretation because changes in environmental quality or health often will themselves change the real income (utility) distribution of society. A valuation procedure that sums individual WTP does not capture individual preferences about changes in income distribution. Another complication is that the value of avoided health risk may differ by type of health event and age. For instance, in one study WTP to reduce cancer was about a third larger than that for a similar chronic, degenerative disease [95]. VSL estimates can be adjusted based on existing health condition or age, or by the use of a value per life-year saved [96]. Use of the value of a statistical life year (VSLY) is very controversial, however, because it implies that age and WTP are proportionally and inversely related, although the literature does not support this assumption. Estimates for children are very limited; however VSLs are generally higher for children [97] and the empirical literature suggests that children's values are approximately twice the value for an adult. WTP measures are theoretically superior to the "supply-side" measures of health damage because they can capture the complete value of health, including pain and suffering.

The hedonic labor market WTP approach relates wage differentials to health risk differences across occupations and industrial/commercial sectors, under the theory that in competitive labor markets, workers in risky jobs should receive wage premiums equal to the value they place on avoiding health risks [98,99]. Such studies can ask workers their perception of health risks to address differences between perceived and actual risk. These studies are numerous and form the foundation for most VSL estimates. However, they are problematic for application to health effects of pollution, because of less directly relevant behavioral contexts and/or the populations. In particular, reducing air pollution may lower some health risks disproportionately for older persons who are not in the labor market. These benefits, furthermore, may be more likely for people with chronic heart or lung disease and may have a delayed effect, all of which would not be captured in the labor market studies.

A small literature of consumer preference studies estimates WTP to reduce health risks from purchases or other actual consumer decisions (e.g., purchase of smoke detectors [100], driving behavior under different speed limits [101]). These studies typically find lower VSLs than other approaches [101]. A difficulty about these studies is statistically separating the health risk-reducing attribute from other valued attributes. A large body of literature applies a hedonic property value approach [102], which provides a revealed WTP for air pollution reductions but is dependent on housing market perceptions about pollution and links to non-health effects.

The stated preference WTP approaches, of which CV and choice experiments are most prominent, are survey methods presenting hypothetical choices (e.g., willingness to pay some amount or prefer one set of attributes over another) to recover preferences for health risk reductions. Results can be sensitive to question wording and ordering, and cognition difficulties when understanding small changes in probabilities are required. However these methods can be molded to a particular population or context. Respondents can be tested for their cognition and understanding of the survey's concepts.

Some of the best known stated preference studies for mortality examine traffic fatalities [103,104] and fewer studies are available for air pollution contexts [105-108]. A CV survey found that WTP was higher when death risk reduction takes place now rather than later in life or if the individual was mentally healthy [109]. Age had a relatively minor effect on VSL, and physical health status had no effect. These results are consistent with those from a study of adults in the US and Canada, which did not find strong evidence that WTP is lower for older persons or for those with chronic heart or lung conditions or cancer [110]. A recent WTP study of three countries also found that VSL is not significantly lower for older populations, however persons admitted to the hospital or emergency room for CVD or respiratory causes had higher VSL [111]. The first study to investigate WTP for increased life expectancy (one year in expectation) added between ages 75 to 85 years found implied VSLs to range from $70,000 to $110,000, but did not provide indication of whether respondents understood the complex scenario, and offered respondents an unrealistically large reduction in risk [106].

Two studies applied choice experiments to examine WTP to reduce risks of chronic respiratory disease [112,113]. Subjects chose between two cities for residence, both preferred to their present city and differing in risk of developing chronic bronchitis or respiratory disease and in one other characteristic: the probability of dying in an automobile accident or cost of living. Several studies evaluated the WTP to reduce cancer morbidity risks [114,115].

Three of the first CV studies for acute health responses used bidding procedures to elicit values for respiratory-symptom days, with average estimates from $5 to $25 depending on the symptom, its severity, and whether a complex of symptoms is experienced [116-118]. CV techniques have advanced since these studies, however they offer consistent ranges of WTP estimates. In one of the few European studies of this type, over 1,000 Norwegians were interviewed to ascertain WTP to avoid various ac ute health effects (e.g., one more day over their usual annual frequency). The values for avoiding symptoms are slightly smaller than those found in older US studies, but the asthmatic values are far larger [119]. A survey of 832 Taiwanese investigated WTP to avoid participants' most-recent episode of acute respiratory illness [120]. Statistical techniques are used to relate these values to the duration and severity of the episode and other variables.

Another approach is the averting-behavior method, which infers WTP by observing and placing values on behavior used to avoid adverse health outcomes. For instance, if someone stays indoors with the air conditioner on because of high air pollution, the added electricity costs might relate to WTP to avoid health impacts. Defensible estimates under this approach require stringent assumptions, and in practice the method is rarely used, particularly in an acute-health context.

#### Quality-adjusted life year

The QALY approach attempts to account for the quality of life lost by adjusting for time "lost" from disease or death. This method is welfare-theoretic only under very restrictive assumptions, so it is difficult to conceptualize the significance of any particular QALY score. The estimates may be very insensitive for distinguishing among different severities and types of acute morbidity. See the recent Institute of Medicine report [121] for a full review of this approach as it could be applied in a regulatory, cost-benefit analysis setting.

A QALY analysis of USEPA's Heavy Duty Engine/Diesel Fuel regulations found that for situations in which mortality dominates other health outcomes, QALY and WTP methods can provide similar results [122]. If morbidity and non-health consequences are predominant, results from QALY and WTP analysis may differ. Another use of QALYs investigated over 230 WTP estimates, finding that variation in WTP values is affected by QALY estimates of illness severity, illness duration, income, and age [123]. There also exists literature providing QALY estimates for chronic diseases, for example for various severities of asthma [124].

#### Applications of economic valuation

Valuations of mortality risk reductions associated with environmental policies are usually the largest category of benefits, both among health responses and compared to other attributes. For instance, a USEPA analysis of the Clean Air Act estimated a value of $100 billion annually for reduced premature mortality out of $120 billion in total benefits, compared to costs of approximately $20 billion [7]. European and Canadian studies similarly found that mortality risk dominates analysis of pollution reductions [125,126]. Next to mortality, reductions in the probability of developing a chronic respiratory disease have been estimated to be the most valued, recognizing that values for other types of diseases are sparse. Reductions in acute effects are lower valued.

Table 2 provides a sample of values typically used by practitioners of health benefits analyses from several major studies or models: the USEPA's BenMAP, which is used in Regulatory Impact Analyses of Regulations [7,54]; the ExternE model [125], which is used by the European Union (EU) in its regulatory analyses, taken from its Clean Air For Europe (CAFÉ) Program (AEA) [127]; the Air Quality Valuation Model (AQVM) for Canada [126]; the Australian Bureau of Transport and Regional Economics (BTRE) assessment of transportation-related pollutants in Australia [128]; and a study of the benefits of environmental improvement in New Zealand [129]. Within the table, health values are converted to common, comparable currency using purchasing power parity (PPP) and constant 2000 dollars. The WTP for reducing risks of mortality and chronic morbidity is expressed, for convenience, as VSL and the value of a statistical case (VSC) of chronic disease. This term is merely shorthand for the WTP for a given risk reduction divided by that risk reduction. This relationship is useful because VSLs or VSCs can be multiplied by estimates of the "lives saved" or "chronic cases saved" to obtain benefits.

**Table 2:** Sample of typically used values for PM-related health impacts (mean estimates) ($2000 PPP-adjusted [197])

**Health Effects**	**US**	**EU**	**Canada**	**Australia**	**New Zealand**
*Mortality:*			1,042		1,296,552 (premature death) [198]
VSL: Adults	6,300,000	2,247,191	3,480,000	1,439,394	1,717,241 (1,724,138) [198]
VSL: Children	2 × adult	4088764 (infant)			
VSLY		134,831		70,455	118,621
*Morbidity:*				1929.55 (average cost/separation) [199]	
Morbidity: children	2 × adults				
Chronic bronchitis	340,000	213,483			
Chronic asthma	39,000				
Respiratory hospital admission	14,000	2,247	1,032		2,069
CVD hospital admission	21,000	2,247	1,052		2,759
Emergency room visit	300 (asthma)		541 (respiratory) 562 (CVD)		
Doctor's visit		60			
RAD	106	92 (working age) 78 (young, elderly)	22		53
MRAD	50	43			
Acute respiratory symptom	3–24				
Use of respiratory medication		1.12			
Asthma day	32–74	43	15		
*References:*	[54]	[127]	[200]	[128]*	[129]

Note: *: VSL derived from population-weighted values in the Australian Bureau of Transport and Regional Economics (BTRE) assessment, Table 3 [128]. Population data from the Australian Bureau of Statistic.

The table shows a fairly wide range of VSL values, with the highest in the US. Rank ordering of values across the other health endpoints is very similar across studies, although some different sets of health endpoints are considered and there are many blank cells (i.e., categories for which information is unknown or not incorporated) outside of the US and EU. The relatively close agreement between the US and EU likely results from reliance on a common pool of studies, results, and interpretations as well as the social cost of electricity studies in the US and the ExternE effort in Europe, which benefited from close collaboration between the participating researchers in both efforts [130,131]. In addition, the Canadian studies were informed by the AQVM developed by researchers active in the US social costing debate [132].

#### Credibility of economic valuation estimates

We evaluated economic valuation methods on three criteria: (i) the degree to which methods are based on preferences for such health improvements, which we took to be in agreement with welfare economics principles; (ii) the number of studies following the technique, which is an imperfect measure of degree of consensus and attractiveness of the technique to researchers; and (iii) additional major limitations, serving to capture other issues, such as data shortcomings. Based on this admittedly subjective judgment, we then rated the reliability of the different approaches from *A *(very reliable) to *D *(unreliable). The assessment is intended to provide comparison among approaches, rather than an absolute assessment of accuracy.

As a first step of the evaluation, we compared theoretical predictions and empirical results of economic valuation studies for mortality (Table 3). Under the theoretical framework, WTP should increase with the size of the risk change. The life cycle model also implies lower WTP when risk change is further in time. Persons facing higher baseline risks should have higher WTP for a given risk reduction (the "dead anyway" effect) [133]. Higher incomes or wealth should relate to higher WTP. With borrowing against future earnings, the relationship between WTP and age should be an inverted U-shape according to life cycle models. Finally, these models do not make a prediction regarding health status.

**Table 3:** Theoretical predictions and empirical results of studies estimating value of mortality risk reductions. Source: Hammitt and Graham (1999) [103]

**Study**	**Size of Risk Change**	**Future Risk Change**	**Baseline Risk**	**Income (or proxies)**	**Age**	**Health Status**
Life cycle model: Theory	+, proportional	-	+^a^	+	-^b^, + then -^c^	indeterminate
Empirical Studies						
Compensating Wage	+	N/A	-^d^	+	-	N/A
Other Revealed Preference	+	N/A	Unknown	+	+	N/A
CV	+, not proportional	-	Varies	+	+ then -, 0, -	No effect, +

a. Small "dead anyway" effect: Higher value to benefits while alive than for a bequest [133].

b. With borrowing against future earnings.

c. Inverted U with no borrowing.

d. Self selection by risk tolerant workers

These theoretical predictions are not always matched by empirical results, and Table 3 demonstrates that no simple consistent relationship exists between WTP for mortality and other factors listed, other than income. This could be due to differences in the underlying approaches used to solicit results, or indication of a more complicated system (e.g., age's impact on VSL may further depend on other factors). Our subjective evaluation of the valuation methods for mortality, chronic morbidity, and acute morbidity are provided in Tables 4, 5, and 6, respectively. No single method is fully satisfactory. Due to the array of methods available for estimating the economic impact of health and the limitations of any single approach, we recommend the application of multiple methods.

**Table 4:** Credibility ratings for approaches to valuing changes in the risk of mortality

	**Criteria**			
		
**Approach**	**Welfare Theoretic (Y/N)**	**Numbers of Studies (Many/Some/Few)**	**Other Limitations**	**Rating**
Human Capital	N	M (not recent)	Undervalues non-workers	D
COI	Not usually; in principle could be if separate estimates available for pain and suffering	M	Usually underestimates	C
Revealed preference: Hedonic Labor Market; others	Y	M	Inappropriate commodity/Population sampled	B
CV and choice experiments: health	Y	S	Hypothetical; hard to understand small probability change	B
QALYs	N (except under very restrictive conditions)	M	Monetization arbitrary	C

**Table 5:** Credibility ratings for approaches to valuing changes in the risk of chronic morbidity

	**Criteria**			
		
**Approach**	**Welfare Theoretic (Y/N)**	**Numbers of Studies (Many/Some/Few)**	**Other Limitations**	**Rating**
COI	Not usually; hospitalization; sometimes labor productivity (which is a revealed preference approach)	M: medical cost studies F: labor productivity studies	Pricing medical services can be difficult where medical care is socialized or subsidized	C-B
Revealed preference	Y	Many on injury/accidents; not on morbidity		C
CV and choice experiments: health	Y	F	See above	B
QALYs	Y (under very restrictive conditions)	M	Arbitrary monetization	C

**Table 6:** Credibility ratings for approaches to valuing changes in the risk of acute morbidity

	**Criteria**			
		
**Approach**	**Welfare Theoretic (Y/N)**	**Numbers of Studies (Many/Some/Few)**	**Other Limitations**	**Rating**
COI	No	M	Pricing medical services can be difficult	C
Revealed preference (averting behavior)	Y (under restrictive conditions)	Many for injury and accidents; not for acute respiratory symptoms		C
CV and choice experiments: Health	Y	S	Old methods/studies; some ad hoc estimates; small samples	B
QALYs	Y (under very restrictive conditions)	M	Scores insensitive to severity of acute effects	C

In addition to the issues of credible economic evaluation of the benefits and costs of climate change policies, a central issue in comparing these values is the discount rate applied [134]. Selection of the discount rate, which accounts for differential value of costs and benefits occurring in the far future compared to those taking place in the present or near feature, can greatly alter results of cost benefit analysis, such as for climate change. In fact, a recent disagreement regarding climate change policy analysis by two leading economists centered largely on the use of a different discount rate [11,134]. While some aspects of benefit/cost analysis are well-suited to monetary terms, the issue of an appropriate discount rate carries ethical implications regarding the relative impacts on various populations.

## Discussion

Estimating the ancillary public health consequences of GHG policies is a challenging task drawing upon expertise in economics, emission inventories, air pollution modeling, and public health. However, to date most assessments have focused more heavily on one aspect of the framework (i.e., a portion of Figure 1), whether it be estimation of changes in air pollutant concentrations, health response, or economic analysis (see Table in additional file 1). We have summarized the limitations in the health and economics estimations, however other uncertainties exist for the selection of policy alternatives and estimation of changes in air quality. In spite of differences in approaches, choice of climate change policy, etc., the wealth of evidence from multiple studies provides a broad consensus that ancillary health benefits from improved air quality are substantial, which can be useful information for the policy debate about the scope, design, and timing of climate policy.

Results from current ancillary benefits studies may be underestimates due to unquantified benefits, as only a subset of the health consequences from air pollution have adequate exposure-response relationships [59,135-137]. A USEPA evaluation of the Clean Air Interstate Rule (CAIR) noted numerous unquantified health impacts such as chronic respiratory damage for O_3_, pulmonary function for PM, and lung irritation for NO_x _[135]. The nature of unquantified effects is continually evolving. Some pollution and health relationships considered unquantifiable by USEPA in 1999 [7] have since been identified, such as for acute O_3 _exposure and mortality [51,76-78] and air pollution's association with lung cancer [138,139]. Further some endpoints may be included in one analysis, but regarded as too uncertain for another, perhaps due to a different study location or differences in researchers' judgment. One approach to address health endpoints with uncertain concentration-response functions is to include these effects qualitatively in discussion of unquantified benefits. Another is to incorporate these effects in sensitivity analysis.

Similarly, some economic costs may not be easily quantifiable, even if the health response to air pollution is understood. For example, the USEPA's CAIR analysis identified several unquantifiable costs including employment shifts as workers become reemployed, administration costs in state and federal governments, and some permitting costs [135]. Only a limited number of studies are available regarding the value of children's health, such as several that estimated the cost of children's asthma [140-143]. Valuing reduced mortality risks for newborns or children is challenging because children are generally not the key decision-makers over their own health. Techniques to transfer adult monetary valuations to children have been explored [144].

This work has focused primarily on health benefits from improved air quality resulting from climate change mitigation, however a full assessment of the short-term consequences of climate change policies would incorporate tradeoffs that may in fact be negative or for which the direction of impact is difficult to predict. Policies might alter unemployment rates and income levels, which have been linked to increased suicides [145,146], domestic violence [147,148], depression [149], and mental health [150,151]. The relationships between low income or unemployment and health are not fully understood and somewhat controversial. Still, changes in employment or income from climate policies have the potential to introduce another set of health-related ancillary benefits or costs.

As another example, GHG mitigation might incorporate policies to deter suburban sprawl, which could reduce transportation-related air emissions and thereby improve health in the short-term. However, a fuller understanding of the consequences of such a policy would address changes in population-weighted air pollution exposure, which may be higher in urban areas, as well as urban crime, and other potential impacts from higher population density. Other examples are transition to biofuels, which could have implications for nutrition, or the use of bikes rather than cars for transportation, which would lower air pollution emissions but could potentially also harm health if biking occurred near major roadways, increasing proximity to high pollution at an increased ventilation rate, or could improve health through increased exercise. Thus, while our discussion and most research of ancillary consequences have focused on benefits, a full suite of positive and adverse consequences could exist.

One of the most controversial aspects of ancillary benefits analysis is the valuation of health in non-industrialized countries. Previous Intergovernmental Panel on Climate Change (IPCC) assessments sparked heated debate because they presented non-market values for health improvements that some thought unethically devalued lives in non-industrialized countries. Challenges to economic valuation of health in these regions are described elsewhere [152]. Limited data availability, such as for wages, prohibits application of some approaches. Medical cost information may not reflect social opportunity costs. Hedonic labor market studies, which presume that labor and goods markets are competitive and workers have reasonable information on death and injury risks, may carry more uncertainties in some regions than others. Valuation of the health of various household members, particularly children, may be quite different than in developed countries because of children's more central role in the economy. Rapid economic growth means preferences are changing as well, raising questions about the applicability of indigenous studies several years hence.

A related challenge is differential effects by subpopulations. Epidemiological evidence supports the hypothesis that some segments of the population (e.g., racial or socio-economic groups) face disproportionate health burdens from air pollution. Current ancillary benefit analysis does not include separate estimation of health and economic damages by sub-groups or confront issues of environmental justice. Further information is needed on the relationship between air pollution and health and economic valuation methods with respect to subpopulations.

In order to conduct the most robust ancillary benefits analyses, we recommend reliance on the most defensible, transparent methods, even if they are recognized as deficient. Because a variety of approaches are available, none of which are ideal, we recommend the application of multiple methods and extensive sensitivity analysis considering a range of changes in air pollution concentrations, spatial distribution of impacts (if considered), health endpoints, epidemiological concentration-response functions, and economic valuation estimates.

## Conclusion

Overall, though still a work in progress, the present techniques available for the analyses of the ancillary public health costs and benefits are adequate and appropriate for implementation by those comparing the relative merits and overall value of various GHG mitigation policies. Estimates of considerable benefits that remain after a variety of sensitivity analyses can alleviate some concerns regarding limitations of individual methods or assumptions. The short-term public health changes associated with GHG mitigation strategies should be considered as a key factor in the choice of GHG policies.

## List of abbreviations

AQVM Air Quality Valuation Model, BenMAP Benefits Mapping and Analysis Program, BTRE Bureau of Transport and Regional Economics, CAAA Clean Air Act Amendments, CAIR Clean Air Interstate Rule, CO_2 _carbon dioxide, COI cost of illness, CV contingent valuation, CVD cardiovascular, EU European Union, GHG greenhouse gases, MRAD minor restricted activity days, NO_x _nitrogen oxides, O_3 _ozone, PM particulate matter, PM_10 _particulate matter with an aerodynamic diameter ≤ 10 μm, PM_2.5 _particulate matter with an aerodynamic diameter ≤ 2.5 μm, PPP purchasing power parity, QALY quality-adjusted life year, RAD restricted activity days, SO_2 _sulfur dioxide, TSP total suspended particles, USEPA US Environmental Protection Agency, VOCs volatile organic compounds, VSC value of a statistical case, VSL value of a statistical life, VSLY value of a statistical life year, WTP willingness to pay.

## Competing interests

The authors declare that they have no competing interests.

## Authors' contributions

All authors made substantial contributions to the conception and design of this paper, were involved in drafting and revising the manuscript. All authors have approved the final version.

## Supplementary Material

Additional file 1 Studies investigating the air pollution and health co-benefits from climate change policies Click here for file
