# Supplementary material for: Ancillary human health benefits of improved air quality resulting from climate change mitigation
Source: Environ Health. 2008 Jul 31;7:41. doi: 10.1186/1476-069X-7-41 (PMC2519068; doi:10.1186/1476-069X-7-41)
Supplement: Additional file 1 — Studies investigating the air pollution and health co-benefits from climate change policies [file 1476-069X-7-41-S1.doc]

**Table 1.**

**Studies investigating the air pollution and health co-benefits from climate change policies**

| **Aaheim et al. 1999** [153]  *Area and timeframe:* Hungary (5-year period)  *Pollutants:* Total suspended particles (TSP), sulfur dioxide (SO2), NOx, non-methane volatile organic compounds (VOCs)  *Policies:* Several energy policies (e.g., increased energy awareness, public transport, and energy efficiency)  *Methods:* 1. Existing analysis of energy options. 2. Exposure-response functions from epidemiological studies. 3. Western valuation studies with a relative income approach. Monte Carlo simulations to assess aggregated uncertainty. Estimation of the cost of energy policies.  *Results:* Annual health benefits $648 million (US), with a range of $370 million to $1168 million. |
| --- |
| **Aunan et al. 2004** [75]  *Area and timeframe***:** Shanxi Province (2000)  *Pollutants*: PM10, SO2  *Policies:* Six policies to reduce coal use  *Methods:* 1. Estimation of pollution reduction potential for each policy. 2. Concentration-response functions from local studies where available; data from elsewhere otherwise. Assessment of chronic mortality, omitting acute mortality to avoid possible overestimation, based on US studies. Assumption of proportional reduction in pollutant levels and population-weighted exposure. 3. Estimation of the cost of each abatement strategy. Value of a life year (VOLY) using Western studies and surveys of the value of a statistical life (VSL). Cost of illness (COI) and willingness to pay (WTP).  *Results:* Across the six policies, $32.4-120.4 (US) local health benefit/year per ton CO2 reduced. Net cost of CO2 reductions was negative (i.e., net benefit) for all policies using central health estimates. |
| **Burtraw et al. 2003** [23]  *Area and timeframe***:** United States (2000-2010)  *Pollutants***:** NOx, PM10, TSP, SO2, sulfate  *Policies:* CO2 mitigation policies in the energy sector  *Methods:* 1. Electricity market equilibrium model and source-receptor matrices. 2. Concentration-response functions based on US Environmental Protection Agency (USEPA) Criteria Documents. Acute-related mortality for PM10. 3. Assignment of monetary values based on the environmental economics literature, with sensitivity analysis by VSL. |

| *Results:* Total ancillary benefits of $12-14 for a $25 Carbon tax. |
| --- |
| **Changhong et al. 2006** [154]  *Area and timeframe:* Shanghai (2000-2020)  *Pollutants:* SO2, PM10, NOx  *Policies:* Coal use cap of 50 million tons/yr. Second approach with coal use capped at 45 million tons/yr.  *Methods:* 1. MARKAL energy and economic model  *Results:* Slowed growth of CO2 emissions coincides with co-benefits of 53% reduction in SO2 emissions and 35% reduction in PM10 emissions. |
| **Cifuentes et al. 2001** [12, 42]  *Area and timeframe***:** Mexico City, São Paulo, Santiago, New York (2001-2020)  *Pollutants***:** O3, PM10  *Policies:* GHG mitigation scenario, largely based on estimates for Chile using readily available technologies in energy, transport, residential, and industrial sectors.  *Methods:* 1. Assumed 10% reduction in pollution levels. 2. Concentration-response functions from local studies where available. Population growth factor assumed for each city. Chronic and acute mortality considered.  *Results:* Averted health outcomes include 64,00 deaths, 65,000 cases of chronic bronchitis, and 37 million person-days of restricted activities in 2020. |
| **Dessus and O’Connor 2003**[155]  *Area and timeframe***:** Santiago (2010)  *Pollutants*: PM10, SO2, NO2, carbon monoxide, O3  *Policies*: Various CO2 reductions  *Methods:* 1. Dynamic computable general equilibrium economic model and dispersion coefficient model linking emissions to concentrations. O3 estimated with quadratic function of VOC and NO2 concentrations. 2. Concentration-response functions based largely on an existing review of epidemiological literature. 3. Economic valuation using WTP where possible, and otherwise using COI, transferring results from US studies.  *Results:* 20% CO2 reduction results in no net welfare loss. 10% CO2 reduction closer to optimal benefits. |
| **Dudek et al. 2003** [156]  *Area and timeframe:* Russia (2008-2012)  *Pollutants:* TSP, SO2, NOx  *Policies:* Scenario with market reforms and emissions trading. Second scenario using old technologies, increased electricity exports, and negative changes in the energy balance.  *Methods:* 1. Economic development model with air pollution projections to estimate changes in pollution emissions. 2. Baseline burden estimates of mortality from air pollution from previous work. Changes in mortality calculated in relation to changes in emissions. 3. Benefit-transfer methods with VSL. |

| *Results:* 30,000 to 40,000 lives saved annually by 2010 with first scenario compared to the second scenario. |
| --- |
| **Mazzi and Dowlatabadi 2007** [157]  *Area and timeframe:* United Kingdom (2001-2020)  *Pollutants:* PM10, PM with an aerodynamic diameter < 2.5 μm (PM2.5), other pollutants mentioned through discussion.  *Policies:* Climate change policy to reduce CO2 emissions through transition from petrol to diesel cars from 2000 to 2020.  *Methods:* 1. Modeling of annual diesel vehicles estimated through additions and subtractions of registrations, and subsequent changes in emissions. 2. Concentration-response functions for mortality and hospitalizations from individual epidemiological studies and coefficients previously developed through multiple time-series studies.  *Results:* An additional 90 deaths/year and 32 hospitalizations/year from diesel policy. |
| **McKinley et al. 2005** [49]  *Area and timeframe:* Mexico City (2003-2020)  *Pollutants:* O3, PM10  *Policies:* Five control measures  *Methods:* 1. Emissions factors and activity levels for each technology. O3 levels estimated through isopleths. Changes in PM10 estimated with reduction factors applied to primary pollutants. 2. Population-weighted exposures combined with concentration-response functions. Use of epidemiological studies based in Mexico City where available. 3. Estimation of investment costs and fuel savings. Estimation of health costs avoided with direct health costs, productivity loss, and WTP.  *Results:* 100 deaths, 700 cases of chronic bronchitis, and over 500,000 minor restricted activity days (MRAD) avoided annually, with about $10 million (US) benefits each year. |
| **Syri et al. 2002** [158]  *Area and timeframe:* Finland (1998-2020)  *Pollutants:* SO2, NOx, VOC, PM2.5  *Policies:* Two scenarios from planned national climate change mitigation measures  *Methods:* 1. FRES emissions scenario model. O3 concentrations estimated through photochemical trajectory model. For other pollutants, estimation of emissions levels.  *Results:* Emissions of NOx reduced 10-12%, SO2 28-32%, PM2.5 3-6%. |
| **van Vuuren et al. 2006** [159]  *Area and timeframe:* Europe (1990-2010)  *Pollutants:* O3, SO2, NOx, VOC, PM10  *Policies:* Three policies with various trading schemes: domestic action, restricted trade, and normal trade. |

| *Methods:* 1. Linked system of models for climate policy (FAIR), energy (TIMER), and regional air pollution (RAINS) to convert regional GHG reductions to regional pollutant levels. 2. Health impacts not directly assessed. Use of modeling system to estimate reduction in human exposure to harmful pollutants. 3. Economic costs of policies incorporated into modeling system.  *Results:* Cost savings of €2.5-7 billion. Reduced pollution levels, such as 15% for SO2. |
| --- |
| **Wang and Smith 1999** [15]  *Area and timeframe*: China (2000-2020)  *Pollutants*: SO2, PM  *Policies*: 10% reduction in GHG emissions by 2010 and 15% reduction by 2020 through energy efficiency and fuel substitution in residential and power sectors  *Methods:* 1. Gaussian plume model. Various assumptions regarding conversion of emissions to dose and exposure, including time spent outdoors. 2. US-derived concentration-response functions  *Results:* 1,500 to 530,000 deaths averted by 2020, depending on policy scenario and assumptions. |
| **West et al. 2004** [21]  *Area and timeframe:* Mexico City (2010)  *Pollutants:* SO2, NOx, hydrocarbons, PM10  *Policies:* 18 emission control options  *Methods:* 1. Indicators of local pollutants reported by previous studies of emissions control strategies used to construct a database of alternatives. Linear programming to identify least-cost strategies to meet multiple pollutant targets. 2. Health impacts not directly estimated. Reports changes in pollution levels. 3. Estimated cost of control options from previous studies.  *Results:* Identified synergies between local pollutants and GHG strategies, but small benefits for Mexico City. Results indicate that simultaneous planning of urban air quality and GHG policies is more cost-effective that separate management of these issues. |
| **West et al. 2006** [13]  *Area and timeframe:* Global (2010-2030)  *Pollutants:* O3  *Policies:* 20% methane reduction beginning 2010 and sustained to 2030  *Methods:* 1. MOZART-2 model to estimate O3 concentrations. 2. US concentration-response function estimate averted mortality. 3. VSL.  *Results:* About 30,000 deaths prevented globally in 2030, and about 37,000 prevented between 2010 and 2030. Benefit of ~$240 per ton of methane, which exceeded the marginal cost of methane reduction |
